# Quantum algorithms for geologic fracture networks

**DOI:** 10.1038/s41598-023-29643-4

**Published:** 2023-02-18

**Authors:** Jessie M. Henderson, Marianna Podzorova, M. Cerezo, John K. Golden, Leonard Gleyzer, Hari S. Viswanathan, Daniel O’Malley

**Affiliations:** 1 Los Alamos National Laboratory, Los Alamos, NM 87545 USA; 2 Southern Methodist University, Dallas, TX 75205 USA; 3 Theoretical Division, Los Alamos National Laboratory, Los Alamos, NM 87545 USA; 4 Department of Computer Science and Joint Center for Quantum Information and Computer Science, The University of Maryland, College Park, MD, 20742 USA; 5 Information Sciences, Los Alamos National Laboratory, Los Alamos, NM 87545 USA; 6 Center for Nonlinear Studies, Los Alamos National Laboratory, Los Alamos, NM 87544 USA; 7 Brown University, Providence, RI 02912 USA

**Keywords:** Hydrology, Computer science

## Abstract

Solving large systems of equations is a challenge for modeling natural phenomena, such as simulating subsurface flow. To avoid systems that are intractable on current computers, it is often necessary to neglect information at small scales, an approach known as coarse-graining. For many practical applications, such as flow in porous, homogenous materials, coarse-graining offers a sufficiently-accurate approximation of the solution. Unfortunately, fractured systems cannot be accurately coarse-grained, as critical network topology exists at the smallest scales, including topology that can push the network across a percolation threshold. Therefore, new techniques are necessary to accurately model important fracture systems. Quantum algorithms for solving linear systems offer a theoretically-exponential improvement over their classical counterparts, and in this work we introduce two quantum algorithms for fractured flow. The first algorithm, designed for future quantum computers which operate without error, has enormous potential, but we demonstrate that current hardware is too noisy for adequate performance. The second algorithm, designed to be noise resilient, already performs well for problems of small to medium size (order 10–1000 nodes), which we demonstrate experimentally and explain theoretically. We expect further improvements by leveraging quantum error mitigation and preconditioning.

## Introduction

Simulating flow in geologic fracture networks requires computing certain features—for example, pressure—throughout a discretized model of the specified region. Often, fracture network problems are specified as systems of linear equations, and solving such systems can become computationally prohibitive as system dimension increases^1^. Classical computers can thus solve large systems only when information is removed from consideration. Coarse-graining is one technique for reducing system size. Originally developed to model multi-scale biochemical systems, it has become an oft-used means of simplifying linear systems, including in the geosciences^2–4^. Specifically, the coarse-graining technique of upscaling can be accurately applied to geological problems involving spatially-large, materially-homogeneous regions. The technique combines mesh nodes and assigns them an averaged, or upscaled, permeability or other geological feature, losing mesh resolution but, in this context, still preserving approximately accurate solutions^5^.

Fractures exist over a range of at least $10^{-6}$ to $10^4$ m, and the computational requirements involved in completely solving systems comprising over ten orders of magnitude quickly become prohibitive. Such systems also cannot be accurately upscaled because the information thereby lost pertains to small fractures that ought not generally be neglected. Collectively, such fractures can radically transform the network topology, including by possibly pushing the network over a percolation threshold. The small fractures can collectively contribute a significant amount of surface area, enabling stronger interaction between the fractures and the rock matrix, potentially providing complete connectivity that would not otherwise exist in a region^6^. Therefore, fracture network problems cannot be classically solved in their entirety, nor can they be accurately solved with upscaling, making simulation of fracture systems one of the most challenging problems in geophysics^7–10^.

**Figure 1:**
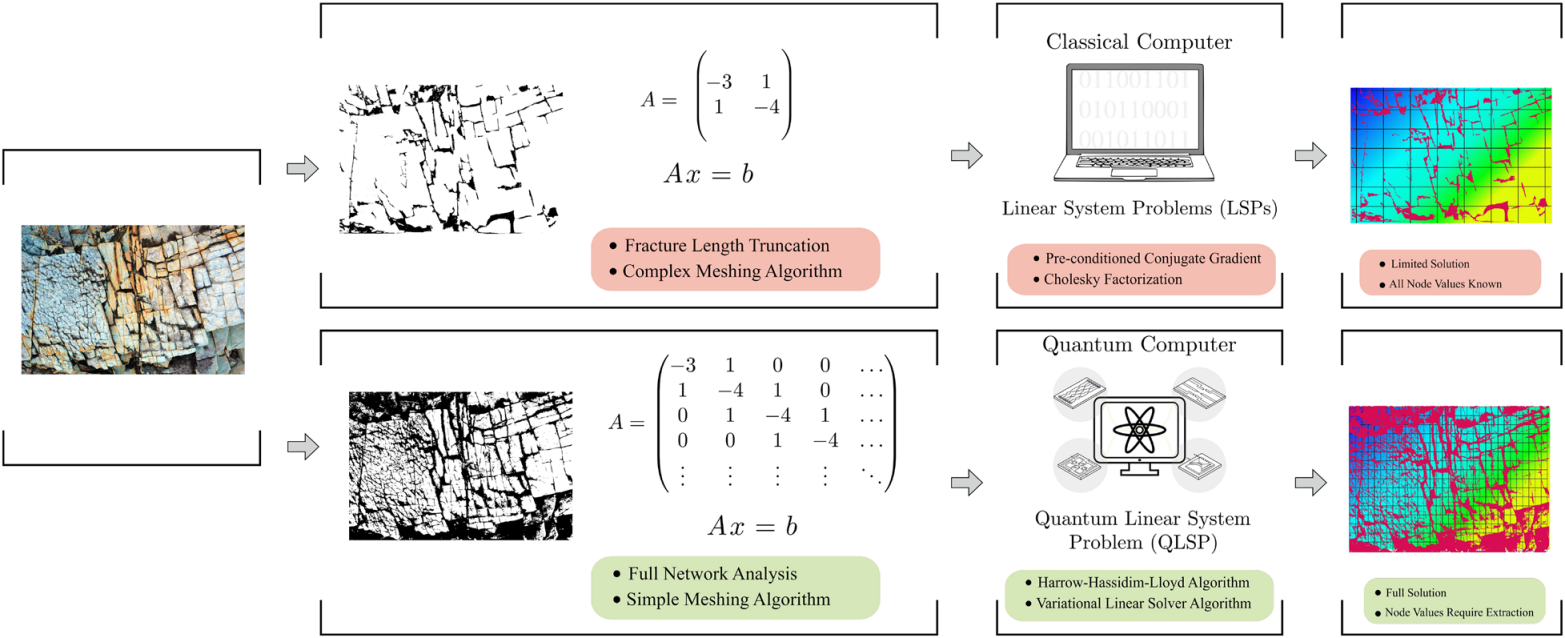
Schematic workflow for applying classical and quantum algorithms to fracture flow problems. Discretizing fracture systems on classical computers involves reducing the computational cost by truncating the fracture network to exclude small fractures. This setting-aside of information provides a solution that does not accurately reflect all flow. Conversely, quantum computing has the potential to solve large, complete fracture systems given properties of quantum mechanics and algorithms designed to take advantage of those properties.

Thus, accurate geologic flow models should include fractures at the entire range of scales. While advanced meshing techniques^11^ and high-performance simulators^12^ allow inclusion of increased fracture range, even such sophisticated approaches do not make it possible to model the full fracture scale. So, as illustrated in Fig. 1, classical approaches to geologic fracture problems depend upon upscaling that neglects information which can dramatically affect the solution.

By contrast, quantum algorithms provide efficient solutions for solving large linear systems that could include the entire scale of geologic fractures^13^. Properties of quantum computing are fundamentally different than classical counterparts, theoretically permitting the solution of classically intractable problems^14–16^. Among other benefits, quantum computers store solutions as a vector, $\psi$, containing $2^n$ elements, where *n* is the number of qubits (or quantum bits)^16^. A quantum computer can thereby solve vast systems of equations with a relatively small number of qubits: *n* qubits allows for solving a system with $2^n$ variables^13^ . Consider a straightforward example involving a cubic fracture domain comprising 1-km and employing a 1-cm resolution. Given $10^5$ centimeters to a kilometer, simulating this region would require $(10^5)^3=10^{15}$ nodes. While a classical computer would thus require $O(10^{15})$ bits, a quantum computer would require only $O(log_2(10^{15}))\approx O(10^1)$ qubits.

This article illustrates using quantum algorithms to solve fracture flow linear systems problems (LSPs) for which upscaling is not appropriate. We introduce two algorithms and provide proof-of-concept application using IBM’s suite of quantum devices. We consider problems formulated as a numerical discretization of $\nabla \cdot (k \nabla h) = f$, where *k* is the permeability, *f* is a fluid source or sink, and *h* is the pressure to be computed. This discretization results in a linear system of equations $(A{{\textbf {x}}}={{\textbf {b}}})$, where *A* is a matrix, and ${{\textbf {x}}}$ and ${{\textbf {b}}}$ are vectors. The solution, ${{\textbf {x}}}$, represents the pressure at each of the discretized nodes, and quantum algorithms prepare a normalized vector proportional to this solution.

Before outlining the remainder of the paper, we make three observations regarding the problems solved and their solutions. First, we note that the problems do not reflect actual geologic data, but are rather generated to represent hypothetical fracture networks that define the *k* in the above partial differential equation. (See “Algorithms for the fault-tolerant era,” “An algorithm for the near-term era,” and “Variational linear solver approach” for details.) We chose this method of generating problems to both keep them sufficiently straightforward for today’s still-developing quantum hardware, and to guarantee that we could solve the problems with classical computers, so that we would have known solutions against which to compare the quantum computers’ results.

Second, we briefly discuss the practicality of a normalized solution from quantum algorithms for solving linear systems. The applicability of such a solution depends upon the physical meaning of the linear system, and there thus may be systems for which a proportional (i.e., normalized) solution is not useful. Fortunately, this is not an issue for fracture network problems, in which a proportional solution is often sufficient, because the goal is to understand relationships between node properties, and not necessarily exact values for those properties. For example, when understanding the direction of fluid flow between two designated nodes or regions in the domain, it is important to understand which node or region has higher pressure, but not necessarily the exact value of the pressures. Thus, in this work, we consider obtaining proportional solutions from the quantum algorithms, and do not consider obtaining a re-scale factor. That said, obtaining such a re-scale factor is possible, and future work could explore the most straightforward means of doing so. For example, the re-scale factor could be computed by averaging the fixed-pressure boundary conditions and comparing this to the average of the normalized pressure at those nodes, as computed by the quantum computer.

Third and finally, we note that obtaining this solution from a quantum computer works differently than from a classical machine^16^. Upon quantum algorithm completion, the entire solution is not readily available, and generally requires exponential time to obtain^16–18^. This is no issue for applications in which the goal is not to *know* the entire solution, but is instead to completely *solve* the problem, such that any portion of the solution that a user obtains is accurate. Fortunately, fracture networks present just such a situation; ordinarily, we are interested in the pressure at a small, fixed number of nodes on the computational mesh, such as the nodes corresponding to a well location. Rather than extract the pressure at all nodes from the quantum computer, we need only obtain the pressures at nodes corresponding to the area of interest. Furthermore, fracture flow problems can be specified in such a way that the complexity required to obtain information about multiple nodes’ pressures is reduced. A procedure that we term ‘smart encoding’ allows obtaining the aggregated pressures of a series of nodes at the computational cost of a single node (see Sec. XB online for further details.)

The paper proceeds as follows. “Algorithms for the fault-tolerant era” first presents two algorithms–the Harrow-Hassidim-Lloyd and Subasi-Somma-Orsucci algorithms—that have proven potential for solving LSPs on error-corrected, or fault-tolerant, quantum computers^13^. Despite the potential for exponential gain in certain cases, the high noise levels of current hardware result in poor performance^19–22^. “An algorithm for the near-term era” then turns to algorithms designed for contemporary, noisy intermediate-scale quantum (NISQ) computers^23–26^. Specifically, we experimentally illustrate the noise resilience of the Variational Linear Solver algorithm^27^, which provides improved solution accuracy even on available error-prone machines for fracture LSPs of small to medium size (10 to 1000 nodes). We conclude by situating our results and suggesting future improvements.

## Results

### Algorithms for the fault-tolerant era

The first algorithm for solving quantum linear systems problems (QLSPs) was introduced by Harrow, Hassidim, and Lloyd (HHL)^13^. It solves the sparse *N*-variable system $A{{\textbf {x}}}={{\textbf {b}}}$ with a computational complexity that scales polynomially with $\log (N)$ and the condition number, $\kappa$, of the matrix *A*^13^. This provides an exponential speedup over the best classical approaches when $\kappa$ is small, such as when an effective preconditioner is used. However, the quantum circuit requirements of HHL—when applied to problems of even moderate size—are well-beyond the capabilities of currently available quantum hardware^28^. This is largely because HHL utilizes complex subroutines, such as Quantum Phase Estimation, which require qubits that operate with almost no quantum noise or error. On NISQ hardware, HHL is thus impractical for systems of interest; the largest system solved to date using HHL is of dimension $16 \times 16$^29–34^. Once large fault-tolerant quantum computers are developed, the exponential speedup offered by HHL (and variations/improvements thereon) could play a critical role in advancing subsurface flow modeling.

In the interim, progress in QLSP algorithms has occurred in two directions. The first is to tailor QLSP algorithms to the strengths and weaknesses of current NISQ computers, such as the algorithm we present in “An algorithm for the near-term era” does^27,30,35–37^. The second is to design algorithms that are still intended for fault-tolerant computers, but which do not rely on as many complex subroutines as HHL and thus may perform adequately on NISQ devices^38–41^. One such example is the adiabatic approach of Subasi, Somma, and Orsucci (SSO)^38^. This approach requires only a single subroutine, known as Hamiltonian simulation, while still offering the equivalent quantum speed-up of HHL.

**Figure 2:**
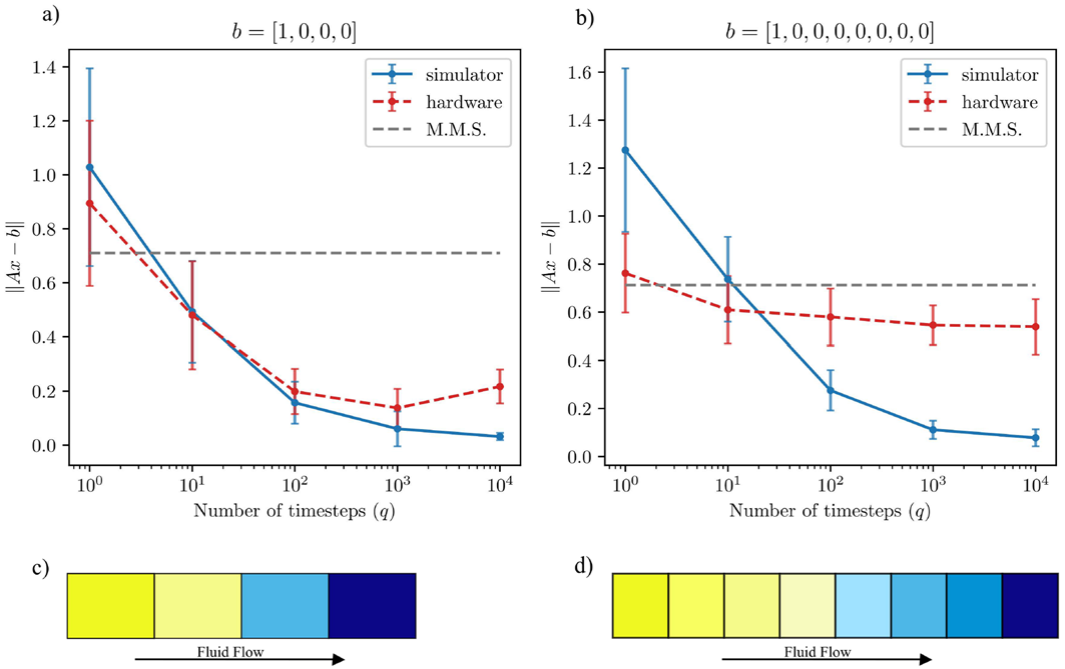
Solving 4-node and 8-node fracture systems using the SSO algorithm. Subfigure (**a**) presents results for a one-dimensional grid of 4 nodes, while subfigure (**b**) does the same for a grid of 8 nodes. Each subfigure presents the maximum, minimum, and average error in 75 runs on either IBM’s quantum simulator (solid blue line) or the ibmq_rome quantum computer (dashed red line). The error is plotted against the number of iterations, *q*, used. The figures also include a dotted gray line illustrating the error that would be achieved if the returned ‘solution’ from the quantum computer was the result of the maximally-mixed state. As described in the main text, this serves as a benchmark for assessing the quality of the solution SSO achieved. Subfigures (**c**,**d**) present cartoons of the problems solved: each color indicates a unique node pressure, with fixed pressures of 1 and 0 on the boundaries.

Before embarking on the purely NISQ-oriented approach of “An algorithm for the near-term era,” we tested the SSO algorithm on a collection of very simple subsurface flow problems to assess how well current hardware could handle one fault-tolerant algorithm. As described in “Introduction,” the problem was to compute pressures of a one-dimensional grid of either $N=4$ or $N=8$ nodes. (See subfigures (c) and (d) of Fig. 2 for a cartoon visualization.) Pressures on the boundaries were fixed, and the answer to the QLSP encoded the internal pressures.

The computational complexity and resulting accuracy of the SSO algorithm depend upon a unitless, user-defined parameter, *q*, which is connected to how long the algorithm is allowed to run. In particular, the complexity and depth of the circuit solving the LSP are linear with *q*^38^. We showed that—up to a point—the algorithm returned better results as *q* increased; for both $N=4$ and $N=8$ problems running on a noiseless quantum simulator, the error $\Vert A{{\textbf {x}}}-{{\textbf {b}}}\Vert$ approached 0 for $q=10^4$. On the quantum hardware, the average error after an equivalent time was approximately 0.21 for an $N=4$ problem and 0.54 for an $N=8$ problem. (Note that for these problems, $\Vert {{\textbf {b}}}\Vert =1$).

Figure 2 illustrates these results and two noteworthy points. First, the $N=8$ problem exhibited a clear limit to how much the hardware results would improve with increasing *q*. Indeed, despite increasing *q* by four orders of magnitude, the average error when run on the quantum hardware decreased by only about 0.2. This suggests that—on NISQ-era devices—SSO’s utility is limited even for problems with as few as 8 nodes.

Second, Fig. 2 compares the errors achieved on quantum simulators and hardware to the error when obtaining a result from a quantum state known as the *maximally-mixed state*. This comparison contextualizes the quality of the errors achieved by SSO, because the maximally-mixed state corresponds to a state where noise has destroyed all information in the quantum system, and thus can be characterized as one of random information. Specifically, for the fracture flow LSPs we solved, obtaining a result from a quantum computer in the maximally-mixed state is equivalent to obtaining *any* of the possible states with equal probability. (In other words, a result from a quantum computer in the maximally-mixed state is a ‘solution’ chosen at random from a uniform distribution of all possible solutions.) Such a ‘solution’ is thus not meaningful, because any accuracy is due to randomness, and not to the performance of the SSO algorithm.

Figure 2 illustrates that the SSO algorithm offered very little improvement upon such a randomly-determined solution. For example, in the $N=8$ case, the hardware results offered an improvement of just about 24%: the result from the maximally-mixed state had an error of 0.71, the quantum hardware achieved an average error of 0.54, and so the improvement due to SSO was solely $\frac{0.71-0.54}{0.71}=0.24$.

The fact that SSO’s performance on such small problems was so limited illustrates that, although fault-tolerant algorithms like HHL and SSO have significant promise, the noise on contemporary devices is too high for accurately solving even very small problems using these methods.

### An algorithm for the near-term era

An alternative to fault-tolerant algorithms are those designed to operate in the NISQ regime, often by leveraging robust classical computing alongside quantum hardware. Variational Quantum Algorithms (VQAs)^23,24,26^ encode a task of interest—in our case, solving a linear system—in an optimization problem. In these algorithms, the classical computer steers the optimization process while the quantum computer computes a cost function, which is being optimized. The goal is to train a parameterized quantum circuit such that the parameters minimizing the cost function are also those that cause the circuit to compute the solution to the problem of interest. There are multiple approaches to solving the QLSP in near-term devices^27,35,36^; we focus on the Variational Linear Solver (VLS) algorithm of Ref.^27^. The VLS algorithm trains parameters in a quantum circuit such that, when a cost function is minimized, the solution encoded by the trained circuit is proportional to the solution ${{\textbf {x}}}$ of the LSP.

We employed the VLS algorithm to determine pressures at each node in a discretized model of the subsurface. With VLS, we can currently tackle much more complex problems than we solved with the SSO algorithm. The problems we considered contained a pitchfork fracture with up to 8192 nodes in the discretization.

Before discussing these problems and VLS’ performance, it is worth clarifying the benefits of applying VLS to linear systems such as fracture network problems. As a variational algorithm, VLS is inherently difficult to classify when it comes to complexity^24,27^. Consequently, it is difficult to mathematically prove a quantum advantage. However, despite the non-convex nature of variational algorithm optimization landscapes, there exist several theoretical results which suggest good performance. These include the absence of vanishing gradients, and the ability to over-parametrize the quantum circuit, which transforms the landscape into a quasi-convex one, allowing the derivation of convergence guarantees in some cases^22,42,43^. Moreover, several quantum-aware classical optimizers have been developed with the goal of furthering variational algorithm performance^44^.

In addition to those theoretical results, empirical studies have shown VLS to have “linear scaling in $\kappa$, logarithmic scaling in $1/ \epsilon$, and polylogarithmic scaling in *N*,” where $\kappa$ is the condition number of the *A* matrix in the linear system, $\epsilon$ is the desired error, and *N* is the dimensionality of the square system^27^. These complexity results are derived from numerical simulations, and suggest that VLS potentially provides an efficiency improvement on the polynomial complexity of classical linear solvers in *N*, while also exhibiting usability on near-term, increasingly-available quantum devices. This motivates our work, as we seek to show that VLS can be accurately applied to linear systems representing fracture flow problems. Therefore, while the quantum advantage of VLS is difficult to theoretically specify, we identify two benefits of applying it to fracture flow problems: first, VLS is a near-term algorithm that has been empirically shown to produce accurate results when solving linear systems problems on contemporary quantum hardware. Second, variational quantum algorithms (including VLS) are similar to other heuristic methods that need to be optimized, such as many machine learning methods. VLS is analogous to ML methods in that—although they may not have theoretical performance guarantees—they may nonetheless provide valuable insights when studied.

#### A $6 \times 8$ domain with a uniform pitchfork

**Figure 3:**
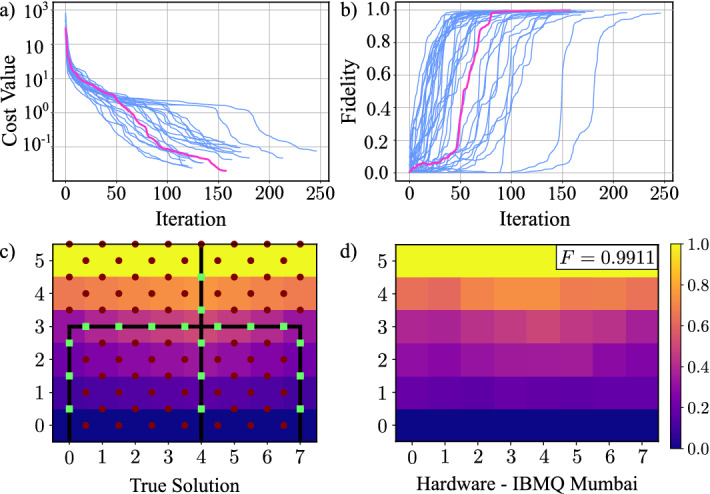
Solving a $6 \times 8$ pitchfork fracture problem using a quantum computer. Subfigures (**a**) and (**b**) illustrate the cost and fidelity per iteration for forty sets of randomly-initialized parameters; the result with the highest fidelity is highlighted. Subfigure (**c**) illustrates the normalized, known, classically computed solution with overlaid permeabilities. The inner $4 \times 8$ nodes are the sought-after pressure values because the top and bottom rows have fixed boundary pressures. The maroon dots illustrate low permeabilities, and the connected green squares illustrate the fracture. Subfigure (**d**) is the solution from quantum hardware (specifically, qubits 0, 1, 4, 7, and 10 of the ibmq_mumbai machine). This solution has fidelity 0.9911, to four figures.

We started with the results of Fig. 3, which illustrates that VLS determined the pressures in a 32-node region with a fidelity of greater than 99%. Fidelity is a common metric for comparing quantum states, and we apply it as a performance measure for a quantum algorithm^16,45–47^. (It is worth noting that there are a number of ways of comparing quantum states^16^, and future work might show that considering alternative metrics provides additional insight into a quantum algorithm’s solution quality, particularly for problems of larger sizes.) Fidelity can be defined as the inner product between two vectors, and thus, fidelity is 1— or 100%—when two vectors have the same direction and proportional magnitude, which equates to a perfect solution in our fracture situation. (Recall that, since the quantum computer produces a solution vector normalized to 1, the output is proportional to the pressure solution.) Conversely, fidelity is 0 when two vectors are orthogonal to each other, meaning an entirely inaccurate fracture pressure solution. Subfigures (a) and (b) illustrate that the VLS training process—in which we simulated the quantum hardware—generated circuit parameters such that a fidelity of 0.9987 was achieved in the best simulation (highlighted in magenta). Furthermore, subfigures (c) and (d) illustrate that noise on quantum hardware did not appreciably damage the solution: when running the circuit with the parameters found via optimization, we achieved a fidelity of 0.9911, only 0.0076 away from the fidelity achieved using a noiseless simulator.

Although this is a very small problem when compared to what classical algorithms can accommodate today, this result is significant because it experimentally illustrates that the VLS approach has some resilience to the noise present in NISQ machines. That in turn suggests why accurate results from quantum computers—even on small problems—are worth exploring. Quantum computing, both algorithmic and physical implementation, is still in its infancy, so, accurately solving proof-of-concept problems like this one is an important step towards understanding how to make use of quantum computing for fracture systems.

#### Larger domains with uniform pitchforks

Success with the 32-node problem led us to consider using VLS to solve larger problems. As predicted, noise affected these solutions more than in the case of Fig. 3 because increasing region size requires larger circuits—including more qubits and more parameterized quantum gates—to encode the problem. Nonetheless, we again found that our solutions were quite accurate: the lowest fidelity was 0.8834 for an 8192-node problem.

**Figure 4:**
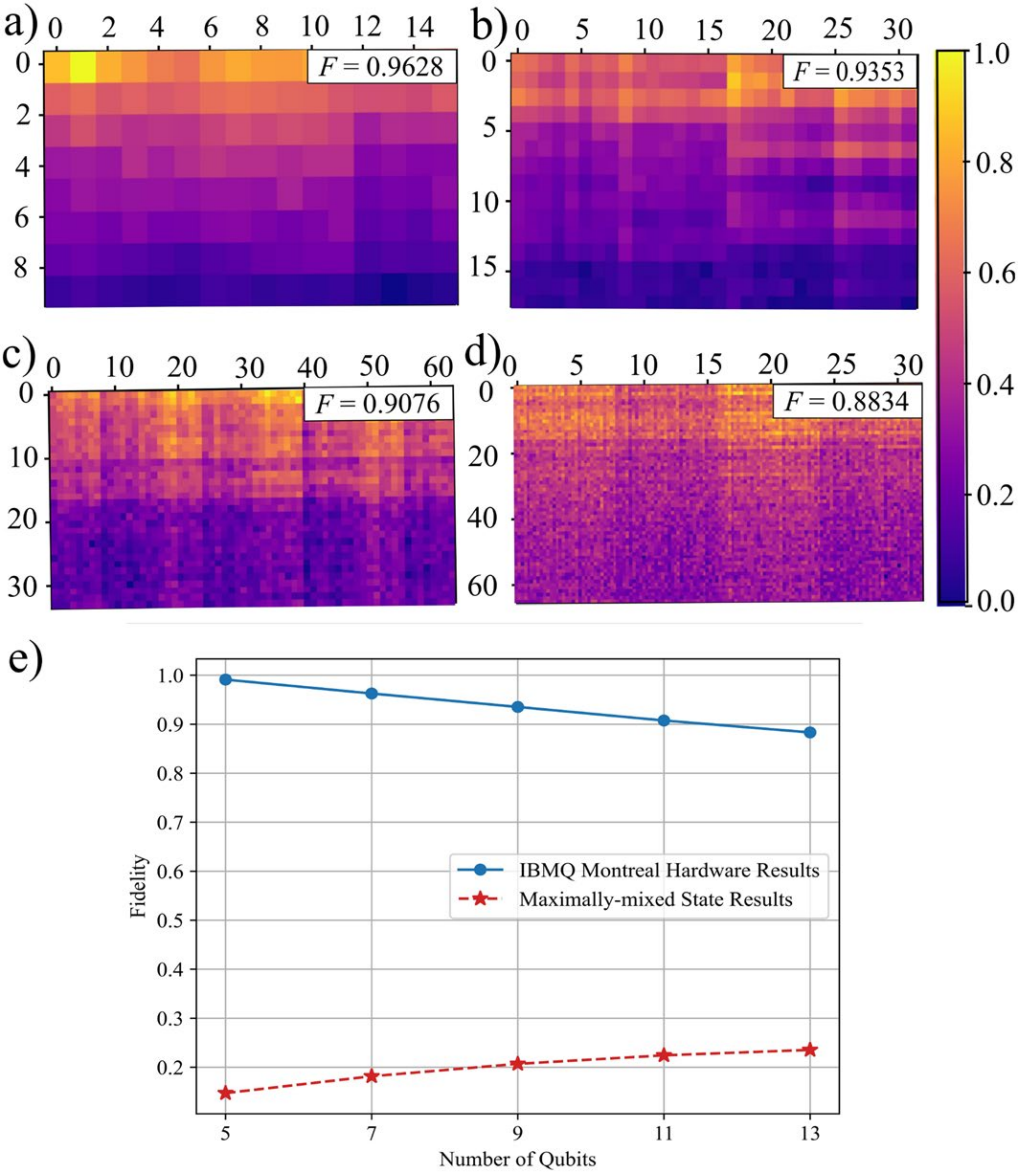
Solving a series of larger fracture flow problems. Subfigures (**a**) through (**d**) are results achieved by running the VLS-trained circuits on the ibmq_montreal quantum computer for 128-, 512-, 2048-, and 8192-node regions. (These correspond to 7-, 9-, 11-, and 13-qubit problems, respectively.) Associated fidelities are 0.9628, 0.9353, 0.9076, and 0.8834. Subfigure (**e**) plots fidelities from the same series of problems (in addition to a 5-qubit problem) alongside the fidelity that would have been achieved had the quantum computer’s prepared solution degraded to the maximally-mixed state. The latter illustrates the fidelity from a result comprised solely of random ‘noise’, thus demonstrating how much better the achieved result on quantum hardware is and experimentally illustrating the noise resilience that is partially proved in Sec. XA, online.

Figure 4 illustrates the details, with subfigure (e) being the most significant result: it indicates that—for all problem sizes considered—we achieved solutions that were significantly more accurate than solutions that had degraded to noise alone. As in “Algorithms for the fault-tolerant era,” we compared the quality of the solution achieved on quantum hardware to a ‘solution’ that would have been the result of the maximally-mixed state. And, as in “Algorithms for the fault-tolerant era,” the maximally-mixed state result is a random solution selected from the distribution of all possible solutions. Unlike with SSO, we found that the quality of VLS’s solution was significantly higher than that from the random solution, even for problems that were larger and more complicated than those solved with SSO. Even the worst fidelity achieved was appreciably above that achieved by a random, noise-only solution: 0.8834 compared to 0.1472.

The performance of VLS on scaled problems was surprising; even as these are relatively small problems, and even as VLS is designed for noisy hardware, we might have seen significantly worse solution quality, as illustrated by Fig. 4. This is because, although VLS offloads some computations onto error-proof classical machines, *any* circuits running on contemporary quantum computers are susceptible to noise. However, quantum algorithms may be less susceptible to noise, if they posses properties that store the relevant information in specific ways, to keep it ‘protected’ from the effects of at least some types of noise. When we found that the quantum hardware’s worst fidelity for scaled problems was appreciably above the associated ‘noise-only’ fidelity, we decided to explore the extent to which the VLS algorithm is noise-resilient^48^. In particular, a type of noise known as *depolarizing noise* affects quantum states by making it more likely that they will end up in the maximally-mixed state. Thus, when we found that VLS solution’s fidelity was far above that of the ‘random’ solution, we mathematically established that the VLS algorithm does have at least some resilience to depolarizing noise. During that process, we also found that that VLS has similarly-limited resilience to what is termed *global dephasing noise*. Proofs for both of these claims are in Sec. XA, online.

It is important to clarify that our proofs are solely a first step—albeit an important one—towards completely understanding the noise resilience properties of VLS. They assume mathematical models of noise that are limited, in the sense that these models do not encompass as many physical situations as can exist. Specifically, the proofs assume that noise is applied to the quantum state at certain specified locations throughout the circuit, when, in reality, noise could occur at any time during the circuit, including coincidentally with application of a gate operation. Thus, our proofs are designed to illustrate that VLS does have properties that protect quantum states throughout the algorithm from certain, limited quantum noise patterns. These proofs, in combination with with the successful empirical results, suggest that further research and empirical evaluation could more completely characterize properties of VLS that offer more expansive noise resilience than the forms which we proved.

## Discussion

Quantum computers promise computational improvement for a wide variety of applications, including—as shown in this work—geologic fracture problems. Although available quantum hardware allows for solving only relatively small problems ($O(10)$ to ($O(10^3)$ nodes), quantum computers are growing and becoming less noisy. Indeed, there is the potential to begin using fault-tolerant algorithms, such as HHL soon^49–53^.

Moreover, the development of quantum algorithms better poised to make use of current hardware means quantum computers may be useful for fracture flow problems before the fault-tolerant era arrives.

The algorithms presented in this article suggest that the future of simulating geologic fracture flow might lie with quantum, and our results show that using those algorithms is no longer a solely theoretical consideration: we can now run fracture problems on quantum hardware and obtain relatively accurate results. Admittedly, these problems are still small, but assuming that the growth and improvement in quantum computers continues as many expect it to—and as it arguably has for the past few decades—we should not be stuck with small problems forever, or even for very long^50,51,54^. Thus, further experimentation is especially necessary in light of opportunities for further accuracy and scaling, including in less-uniform geologic situations that preliminary investigation suggested were more challenging than their uniform counterparts (see Sec. XC online.) Future work should consider tools such as preconditioning^55^, quantum error mitigation^56–60^, ‘smart encoding,’ (see Sec. XB online), problem-specific parametrized quantum circuits^61,62^, both application- and hardware-specific optimization, and rigorous hardware benchmarking to aid in optimal utility of available devices. All of these approaches are expected to offer more accuracy and efficiency on larger and more complex problems, thus further establishing the role of quantum computing in the geologic fracture space.

## Methods

### Adiabatic, fault-tolerant approach

The SSO algorithm^38^ is inspired by the adiabatic theorem in quantum mechanics, which states that a quantum state will smoothly adapt to changes in its environment if those changes are made sufficiently slowly. In the context of linear systems, SSO starts with a quantum state that solves a trivial system of equations, and then slowly changes the system into the more complex one whose solution is sought. SSO changes the system over a discrete sequence of *q* time steps, and the length of each step is chosen at random from a uniform distribution. Increasing the number of steps is equivalent to slowing the change of the system, which increases the accuracy of the final solution.

As described in “Algorithms for the fault-tolerant era,” we used the SSO algorithm to solve a linear system specifying two trivial fracture problems. Both were one-dimensional grids with either $N=4$ or $N=8$ nodes where the left- and right-boundary nodes had fixed pressures. These conditions—along with the discretized equation in “Introduction”—specified the *A* and *b* for the linear system to be solved.

As Ref.^38^ does not provide an explicit quantum circuit implementation of the SSO algorithm, we were limited to creating a unitary matrix representing the net effect of all *q* steps. Quantum gates are mathematically represented by unitary matrices, so a single-unitary implementation of SSO is equivalent to a single, large, custom-generated gate aggregating the effect of all *q* evolutions of the SSO algorithm. Therefore, for given values of *A*, *b*, and *q*, we generated a unitary matrix via the algorithm described in Ref.^38^. Because physical implementations of quantum computers cannot run circuits comprised of arbitrary gates, we then broke down that generated matrix into gates that can be executed on existing devices. To do so, we utilized a variational approach, specifically employing Yao.jl^63^, a Julia library for differentiable quantum programming.

The $N=4$ case, which involves only two qubits, was straightforward because any two-qubit unitary matrix can be expressed in terms of a circuit composed of 3 controlled-not and 7 single-qubit gates^64^. We used the optimization package Optim.jl^65^ to determine the parameter values for the gates to match any given unitary.

The $N=8$ case was more difficult. The shortest known universal circuit for three-qubit interactions contains 138 gates^66^, which is too many for consistently-accurate performance on existing hardware. We therefore employed a machine learning approach across circuits of increasing gate count until we were able to find a circuit that matched the unitary to a high degree. We were regularly able to find circuits with 50 gates (approximately 30 single-qubit gates and 20 controlled-not gates) that achieved at least $99.67\%$ fidelity. Circuits with fewer gates resulted in poor performance.

Once we had obtained circuits that implemented SSO for our fracture systems, we ran them on IBMQ’s suite of quantum computers. Specifically, we used the ibmq_qasm_simulator to simulate performance on a hardware-noise-free quantum device, and then we compared with performance on the quantum computer ibmq_rome.

Quantum computers—and therefore the algorithms that work thereon—are inherently probabilistic. So, most quantum algorithms require running a circuit many times and ‘measuring’ the resulting state each time to establish a probability distribution of states. The probability that each state occurs provides the vector of solutions for the problem that the quantum algorithm sought to solve. Each run/measurement combination is termed a “shot,” and we ran each SSO circuit with 8192 shots on both the simulator and hardware. Using the results, we could then infer the observed value of our sought-pressure solution, ${{\textbf {x}}}$.

Due to the stochastic nature of the algorithm (i.e., randomly-chosen time lengths, *q*), we averaged performance over 75 instances (i.e., distinct time-evolution sequences generated for fixed values of *A*, *b* , and *q*). Figure 2 depicts the results, which are also described in “Algorithms for the fault-tolerant era.”

Finally, we computed the error that would have occurred had the ‘solution’ in the quantum computer degraded to noise alone. We did this by considering the mathematical representation of the maximally-mixed state, which is a state that contains solely noise. The maximally-mixed state can be represented as a density matrix, $\rho$:

1 $$\begin{aligned} \rho = \frac{1}{2^n}I_{n\times n}, \end{aligned}$$

where *n* is the number of qubits^16^. (Note: Throughout “Methods” and X, we use density operators and Dirac notation, both of which are standard notation for mathematically representing quantum circuits. For a thorough introduction to density operators, please see Ref.^16^. For an introduction to Dirac notation, please see Ref.^67^.) When the maximally-mixed state is measured (which can be mathematically represented as projecting the state onto a specified basis), we obtain a result that is equivalent to selecting a pressure at each node randomly from a uniform distribution of all possibilities.

We can illustrate this by considering the probability of measuring a certain 2-qubit state such as that used for the $N=4$ problems. The probability of measuring a given state, *m* from a quantum circuit represented by density operator, $\rho$ is given by,

2 $$\begin{aligned} p(m) = Tr(M_m^\dagger M_m \rho ), \end{aligned}$$

where $M_m$ is the measurement operator for a given basis. (For an introduction to quantum measurement, please see Ref.^16^.) We seek the probabilities of measuring 00, 01, 10, and 11; in our fracture flow problem, each of these probabilities corresponds to the pressure in one of the nodes. For the computational basis, which we used for the results in this paper, the measurement operators, $M_m$ for each of the above possible solutions are $M_{00}=|00\rangle \langle 00|$, $M_{01}=|01\rangle \langle 01|$, $M_{10}=|10\rangle \langle 10|$, and $M_{11}=|11\rangle \langle 11|$. So, when the state of the circuit, $\rho$, is equivalent to the maximally-mixed state for 2 qubits (i.e., a $4 \times 4$ identity matrix with a coefficient of $\frac{1}{4}$), we have that the probabilities for each possibility are given by $\frac{1}{4}.$ We thus see that—for a circuit in the maximally-mixed state—the probabilities of all possible states have been reduced to the same value, meaning the ‘solution’ of the maximally-mixed state contains no meaningful information. Any resemblance to our desired solution, ${{\textbf {x}}}$, is the result of random chance and not the performance of an algorithm. So, to benchmark SSO against the results of random chance, we computed the error that would have occurred had the quantum computer’s returned ‘solution’ been one of random chance alone given degradation to the maximally-mixed state.

### Variational linear solver approach

#### Introduction to VLS

**Figure 5:**
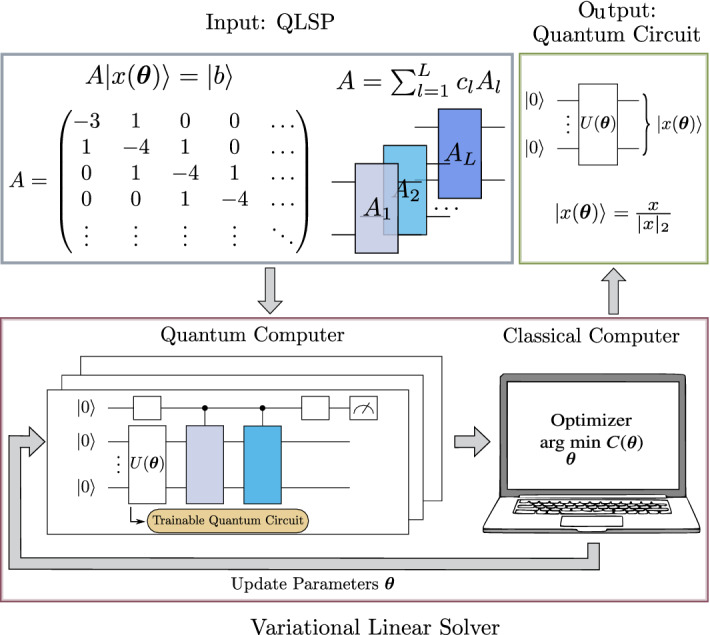
Variational linear solver algorithm. As described in Fig. 1, the algorithm accepts as input an *A* and ${{\textbf {b}}}$ specifying a LSP and minimizes a cost function to create a parameterized quantum circuit that solves the LSP. Specifically, the cost function optimization takes advantage of classical optimization techniques, while the cost function evaluation occurs via either quantum hardware or a classical simulator of such hardware.

As is schematically shown in Fig. 5, the VLS algorithm takes a description of the QLSP (i.e., *A* and ${\varvec{b}}$) as input.

To solve the QLSP, the VLS algorithm trains the parameters $\varvec{\varvec{\theta }}$ in a quantum circuit, $U(\varvec{\varvec{\theta }})$. Figure 7 illustrates the ansatz structure of the quantum circuit that we sought to train with the VLS algorithm. The circuit contains (unparameterized) controlled-*Z* gates and parameterized single qubit rotations about the *y*-axis. Thus, the parameter $\theta _i\in \varvec{\varvec{\theta }}$ corresponds to a trainable rotation angle in the *i*-th rotation such that $\theta _i\in [0,2\pi ]$^27^. We chose this ansatz because it had been used successfully with the VLS algorithm in previous work^27^ and because it is ‘hardware-efficient,’ meaning it uses gates whose structures offer the lowest error-rates available on current NISQ devices.

The $U(\varvec{\varvec{\theta }})$ circuit prepares a trial solution $|x(\varvec{\varvec{\theta }})\rangle =U(\varvec{\varvec{\theta }})|\varvec{0}\rangle$, where $|\varvec{0}\rangle$ is a state in which all qubits are initialized to the easy-to-prepare initial state, $|0\rangle$. To calculate the quality of the resulting quantum state $|x(\varvec{\varvec{\theta }})\rangle$ as a solution to the QLSP, VLS minimizes a cost function $C(\varvec{\varvec{\theta }})$ that quantifies how much each component of $A|x(\varvec{\varvec{\theta }})\rangle$ is orthogonal to $|b\rangle$. It can be verified that the cost function

3 $$\begin{aligned} C(\varvec{\varvec{\theta }})=\langle x(\varvec{\varvec{\theta }})|H|x(\varvec{\varvec{\theta }})\rangle , \end{aligned}$$

with

4 $$\begin{aligned} H=A^\dagger ({\mathbbm {1}}-|b\rangle \langle b|)A, \end{aligned}$$

is minimized if and only if $|x(\varvec{\varvec{\theta })}\rangle$ is proportional to the solution ${{\textbf {x}}}$ of the LSP^27^. Note that, here, VLS maps the QLSP into a problem of finding the ground-state of the Hamiltonian given in Eq. (4).

Once the minimization task, $\mathop {\mathrm {arg\,min}}\limits _{\varvec{\varvec{\theta }}} C(\varvec{\varvec{\theta }})$, is solved, the VLS output is a parameterized quantum circuit that prepares a quantum state $|x(\varvec{\theta _{final}})\rangle$ that approximates ${\varvec{x}}/|{\varvec{x}}|_2$ As mentioned in “Adiabatic, fault-tolerant approach,” obtaining these values requires performing measurements on (i.e., collecting shots from) the state $|x(\varvec{\theta _{final}})\rangle$ to obtain a vector of estimated probabilities that represents a solution to the LSP. Specifically, expressing the solution as

5 $$\begin{aligned} |x(\varvec{\theta _{final}})\rangle =\sum _i\frac{x_i}{\sqrt{\sum _i |x_i|^2}}|\varvec{z}_i\rangle , \end{aligned}$$

where each $x_i$ is an element of $|x\rangle$ and $\{|\varvec{z_i}\rangle \}_{i=1}^{2^n}$ are the elements of the computational basis such that $\varvec{z}_i\in \{0,1\}^{\otimes n}$, then the values $|x_i|^2$ correspond to the pressures at the nodes in the discretized surface. With sufficiently accurate $\varvec{\theta _{final}}$ parameters and enough samples, the vector of estimated $x_i$’s can be brought within a tolerated error of the elements in the desired ${{\textbf {x}}}$.

To assess the quality of a given solution obtained by VLS, there are two approaches that depend upon whether the solution to the problem is known. If the desired vector, ${\varvec{x}}$ is unknown, then the value of the cost function, which takes as input a state $|x\rangle$ generated by a given set of parameters, can be used to evaluate the quality of the final parameters sent as input. In this case, the goal is simply to make the cost function evaluate to as small (i.e., close to zero) a value as possible.

Conversely, if the desired vector, ${\varvec{x}}$ is known—as it was in the experiments we designed—then the quality of parameters can be assessed using a unitless quantity termed *quantum fidelity*. Quantum fidelity is defined as the inner product between two vectors,

6 $$\begin{aligned} F(|x\rangle ,|x_{\text {true}}\rangle )=|\langle x | x_{\text {true}} \rangle |^2, \end{aligned}$$

where $F(|x\rangle ,|x_{\text {true}}\rangle )=1$ if and only if $|x\rangle$ is equal to $|x_{\text {true}}\rangle$ (up to a global unmeasurable phase), and $F(|x\rangle ,|x_{\text {true}}\rangle )=0$ if the two states are orthogonal. Because our goal was to assess the performance of VLS, we solved problems for which we had classically-computed true solutions, meaning we computed the quantum fidelity between the solution obtained by VLS, $|x\rangle$, and a state representing the normalized, true solution $|x_{\text {true}}\rangle$.

**Figure 6:**
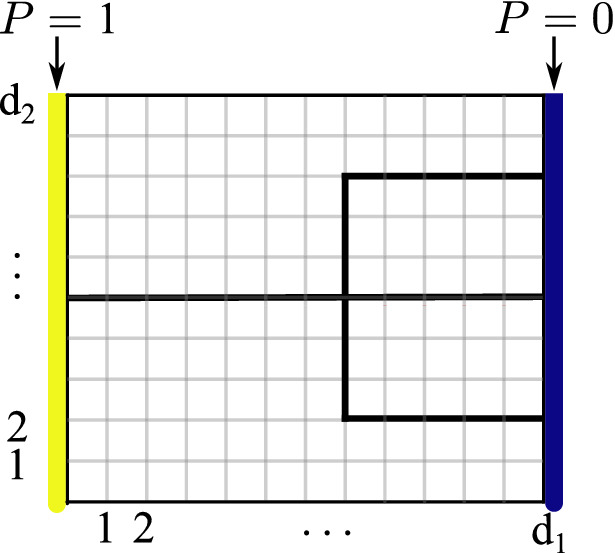
Pitchfork and surface discretization. A cartoon of the subsurface flow situation, in which a region is discretized into $d_1 \times d_2$ nodes. Pressure boundary conditions of 1 and 0 are imposed on the left- and right-hand sides of the region, respectively, and a pitchfork fracture runs through the middle of the region. Each node has a pressure that we seek as our solution.

**Figure 7:**
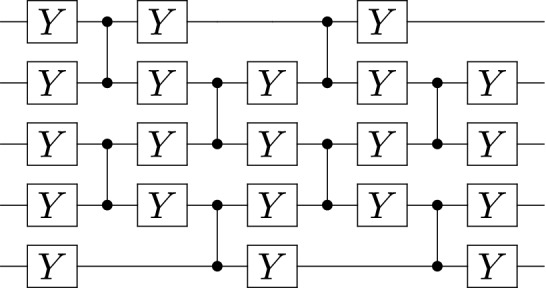
Five-qubit, two-layer ansatz. The pattern for this ansatz is a ‘preliminary’ layer of $R_y$ gates followed by layers with two sets of controlled-*Z* and $R_y$ gates each. Each $R_y$ gate’s angle is one of the tuned parameters.

#### Uniform permeability with $6 \times 8$ region

We first considered a uniformly-permeable pitchfork embedded in a $6\times 8$ grid. As shown in Fig. 6, the subsurface for a given problem needs to be discretized into a grid of size $d_1\times d_2$. For the problem to fit into an *n*-qubit quantum state, the size of the grid needs to be a power of 2, meaning one needs to choose a grid size such that $d_1 d_2=2^n$. In this case, $d_1\times d_2$ did not equal $6\times 8$, but instead $4 \times 8$. Because we imposed Dirichlet boundary conditions, the pressures depicted on the left- and right-most edges of Fig. 6 were fixed at one and zero, respectively. Thus, the solution of interest contained the pressures of only each inner $4 \times 8$ grid node, giving a linear system with *A* of dimension $32\times 32$ and with ${\varvec{x}}$ and ${\varvec{b}}$ of dimension $32 \times 1$. As $2^5=32$, this pitchfork problem was solved using five qubits.

While our approach allowed for the branches of the pitchfork fracture to have varying permeabilities (see Sec. XC online), we first considered a uniformly-permeable pitchfork that had a permeability ten times greater than that of the surrounding surface.

During the training phase of VLS, we ran 40 instances of the algorithm, where the trainable parameters $\varvec{\varvec{\theta }}$ were randomly initialized at each instance. Each of these instances included multiple iterations, where each iteration corresponds to the classical optimizer taking as input the value of the cost function, and producing an updated set of parameters (see Fig. 5). We used Scipy’s optimize package (specifically, minimize) with the conjugate-gradient method^68^ on a five-layer ansatz of the form described in Ref.^27^ and illustrated in Fig. 7. The cost function was evaluated using a classical simulator with shot noise (meaning we used a limited number of shots, and, specifically, $10^8$) but without simulated hardware noise. The cost function was evaluated as described in Ref.^24^. In Fig. 3, subfigures (a) and (b), the cost and fidelity per iteration are plotted for each of the forty instances. The fact that the fidelities per iteration in subfigure (b) converge to one indicates that VLS was able to find the solution of the QLSP. The instance highlighted in purple obtained the highest-fidelity results; after 150 iterations, it achieved a cost function value less than $10^{-1}$, and a fidelity of 0.9987.

We then ran the quantum circuit with the highest-fidelity parameters on quantum hardware and, specifically, qubits 0, 1, 4, 7, and 10 of the ibmq_mumbai machine. These qubits were selected both for their connectivity and relatively low error rates. First, connectivity: it is sensible to select topologically-connected qubits to take advantage of the hardware-efficient structure of the circuit. Otherwise the final circuit would involve additional gates, meaning higher-than-necessary amounts of noise and higher-than-necessary possibility for error. As our goal was to obtain the highest fidelity possible despite the imperfections of existing hardware, we chose qubits that were topologically-connected. Second, error-rate: amongst the connected sets of five qubits available, we chose 0, 1, 4, 7, and 10 because that group avoided inclusion of qubits with high readout assignment errors, high single gate errors, and high controlled-not (i.e., two-qubit) gate errors. We obtained error information via IBMQ’s hardware dashboard; this information changes in real time due to continual calibration of the machines.

We used approximately $10^5$ shots; because the ibmq_mumbai machine has a single-circuit shot maximum of 8192, we ran the circuit 12 times with 8192 shots each time for a total of 98,304 shots. Finally, we permitted Qiskit to perform the maximum number of optimizations allowable by setting the optimization_level flag to three. (Qiskit provides varying automated levels of optimization on a scale of 0—no optimization—to 3—as much optimization as possible^69^).

In Fig. 3, subfigures (c) and (d) illustrate the performance of VLS with the five-layer ansatz and the parameters found in the highest-fidelity instance highlighted in subfigures (a) and (b). Subfigure (c) illustrates the pressure grid corresponding to the normalized, known, true solution of the LSP, as well as the discretized pitchfork fracture as points on the edges of the grid. Subfigure (d) depicts the pressure solution obtained from the quantum hardware. As previously mentioned, algorithms solving QLSPs prepare a solution that is *proportional* to the solution ${\varvec{x}}$ of the LSP, which preserves the relationship between the elements of vector solution ${\varvec{x}}$. We chose to plot the normalized true solution to more clearly visualize that the relationship between solution elements was indeed preserved in the quantum computer’s solution. As is described in “An algorithm for the near-term era,” subfigure (d) indicates that hardware noise did not significantly disrupt the circuit’s ability to compute an accurate solution; the quantum hardware generated a solution with fidelity 0.9911.

#### Uniform permeability with larger regions

We next considered VLS’s scalability on pitchfork fracture problems. For quantum states $|x(\varvec{\varvec{\theta }})\rangle$ of larger dimensions, we can determine the suitability of the solution by minimizing the cost function $C(\varvec{\varvec{\theta }})$ in Eq. (3) as before. However, this direct approach is computationally challenging for circuits with increasingly many qubits. Thus, to simplify the training computation, the minimization of $C(\varvec{\varvec{\theta }})$ may be replaced by globally minimizing a new cost function,

7 $$\begin{aligned} {\tilde{C}}(\varvec{\varvec{\theta }})= 1 - \left| \langle x_{\text {true}} |x(\varvec{\varvec{\theta }})\rangle \right| ^2, \end{aligned}$$

where $|x_{\text {true}}\rangle$ is the solution vector of true pressures. Both Eqs. (3) and (7) achieve minima when $|x(\varvec{\varvec{\theta }})\rangle \approx |x_{\text {true}}\rangle$. Because our goal was to evaluate the performance of VLS, we used only problems for which we could in fact obtain a classical solution to compare against, and this meant that we could classically obtain $|x_{\text {true}}\rangle$ for all of the problems in this article. Thus, we could apply the less-computationally-intense cost function formula above during the training phase.

During the VLS training phase, we once again began with randomly-initialized parameters, and each iteration of the training corresponded to the classical optimizer taking the current value of the cost function to produce an updated set of parameters. Again, we used Scipy’s minimize with the conjugate-gradient method, and, again, we trained with shot noise ($15\times 10^{13}$ shots), but no hardware noise. It is worth noting that the number of shots required to train the circuits such that ${\tilde{C}}(\varvec{\varvec{\theta }}) < 10^{-3}$ (roughly corresponding to fidelities near or above 0.9) increased dramatically for larger problems, which is in part due to the significantly larger circuits that had to be trained. Not only were there more qubits (7, 9, 11, or 13), but because the problems were larger, the circuits also contained more parameterized gates. Experimentation illustrated that a number of ansatz layers greater than or equal to the number of qubits trained circuits well, so we chose the number of ansatz layers to equal the number of qubits.

**Figure 8:**
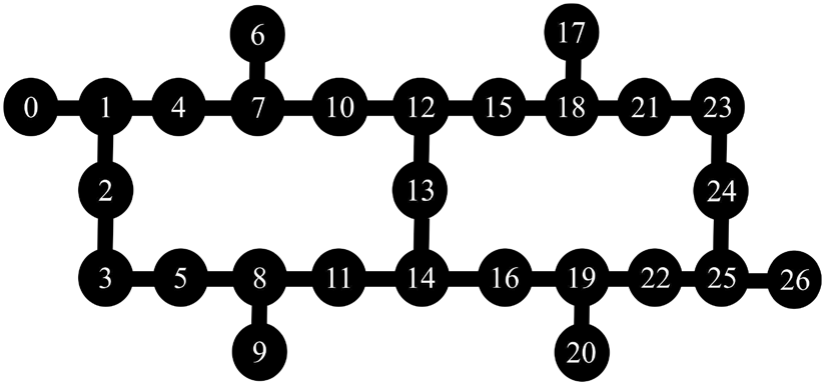
The qubit connectivity of IBM’s Montreal quantum computer.

We then ran the quantum circuit with the highest-fidelity parameters for each of the differently-sized problems on quantum hardware. We again used approximately $10^5$ shots, this time rounding up to 13 runs of 8192 shots each. The qubit selection procedure was more complex because, when selecting five qubits for the smaller problem, it was straightforward to choose a group that avoided the worst-performing qubits. Moving up to even the seven-qubit problem made the selection task more difficult because it was no longer obvious which sets would best reduce error; for example, would it be preferable to include one qubit with very poor performance, or two qubits with better—but still bad—performance? We opted to address this qubit-selection challenge by trying many qubit combinations for each of the $n= 7, 9, 11,$ or 13 qubit problems. Specifically, we used each possible set of *n* qubits in which the qubits were adjacent to each other and did not ‘double-count’ any given qubit. We undertook this procedure for each size of problem on the ibmq_montreal machine, and to help clarify the process, Fig. 8 illustrates the connectivity of ibmq_montreal. Consider the seven-qubit problem: qubits 6, 7, 10, 12, 13, 14, and 16 were a possible qubit selection, but qubits 4, 7, 6, 10, 13, 14, and 16 were not, because the latter would require ‘double-counting’ qubit 7 while determining qubit adjacency.

Figure 4 summarizes our results, illustrating the highest-fidelity pressure solution obtained for the 7, 9, 11, and 13-qubit problems. In particular, the highest-fidelity results occurred for the 7-qubit problem with qubits 3, 5, 8, 11, 12, 13, and 14; for the 9-qubit problem with qubits 0, 1, 2, 3, 5, 8, 11, 13, and 14; for the 11-qubit problem with qubits 5, 8, 11, 14, 16, 19, 21, 22, 23, 24, and 25; and for the 13-qubit problem with qubits 8, 9, 11, 14, 15, 16, 18, 19, 21, 22, 23, 24, and 25. As described in “An algorithm for the near-term era,” subfigure (e) presents results regarding the quality of the solution when compared to a solution containing solely noise.

## Supplementary Information

Supplementary Information.

## Data Availability

The data for generating the figures (excepting those illustrating cartoons/concepts) is available at https://github.com/JessieMHenderson/quantum-geologic-fracture-networks.git. Instructions for generating figures from the data can be obtained from the corresponding author upon reasonable request.

## Code availability

The code used to generate the results is available at https://github.com/JessieMHenderson/quantum-geologic-fracture-networks.git. Instructions for utilizing the code (beyond those contained within the documentation) can be obtained from the corresponding author upon reasonable request.
