# Supplementary material for: Quantum algorithms for geologic fracture networks
Source: Sci Rep. 2023 Feb 18;13:2906. doi: 10.1038/s41598-023-29643-4 (PMC9938886; doi:10.1038/s41598-023-29643-4)
Supplement: Supplementary file 1 — Supplementary Information. [file 41598_2023_29643_MOESM1_ESM.pdf]

X. Supplementary Information

A. Theoretical Results on Noise Resilience

1. Brief Introduction to Quantum Noise

As has been emphasized, current quantum hardware is noisy, meaning it cannot yet sufficiently avoid information loss caused by uncontrolled interactions with the environment or other qubits. This means that the fragile entities we use as qubits (whether those be ions, photons, or microwave-controlled superconducting chips) are changing in such a way that the information we tried to store is damaged [62]. Mathematically, we can represent noise as we can any other operation on a qubit—the only difference is that such noise operations are uncontrolled and determined by qubit-qubit or qubit-environment interaction.

By leveraging an algorithm like VLS, in which a portion of the computation is performed on classical computers, we reduce the amount of quantum noise: because less time is spent on a quantum computer, there is necessarily less opportunity for noise exposure. However, any circuit that is run on a quantum computer is susceptible to noise during that process. So, as long as there is any 'quantum portion' of a hybrid algorithm running on a NISQ machine, exposure to noise is guaranteed. We can mitigate the effects of such inevitable noise by modifying how quantum algorithms store the information most relevant to the ultimate solution. When an algorithm avoids damage from a type of quantum noise through such design features, the algorithm can be said to be *resilient to* that sort of noise.

Before going further, it is worth emphasizing that *resilience* to quantum noise should not be confused with *resistance* to quantum noise, where the latter connotes immunity rather than simply some measure of defense. Demonstrating mathematical resilience to a type of quantum noise does not mean that that sort of noise will have *no* effect on a quantum state because mathematical models always depend upon assumptions. And, as described in Sec. II B, our proofs are only a first step towards completely characterizing the noise resilience of VLS that has been empirically demonstrated. Our assumptions require that the noise occur at certain times throughout the circuit operation, and while those assumptions do allow us to prove VLS has resilience to noise in those cases, we make no claims about even the same types of noise occurring in other locations throughout the circuit.

We do believe, however, that there is more to be shown regarding the noise resilience of VLS. We empirically demonstrated that VLS has far more noise resilience than could be explained by the limited proofs herein. Therefore, future work should further explore the algorithm's noise resilience both mathematically and experimentally: while formal mathematics illustrates that a certain set of experiments was not simply a coincidence, empirical successes show that the assumptions on which the mathematical models were based have sufficient accuracy to derive practically-useful conclusions.

Before providing the theorems and proofs, we provide a brief introduction to the mathematical representation of quantum noise. (For a thorough introduction to quantum noise, please see Ref. [16].) As mentioned previously, quantum states can be represented mathematically using matrices known as density-operators [16]. These provide a more general—albeit sometimes less intuitive—notation than the statevector notation used most often in this article [16]. Density-operators can clearly express the state of a quantum circuit even when we lack complete information about what operations have occurred. Specifically, if some operation will occur with probability p and another with probability $1-p$, density operators can encapsulate these possibilities on the level of circuits and not individual qubits.

A density operator is defined as,

$$\rho = \sum_i p_i |\psi_i\rangle\langle\psi_i|, \tag{8}$$

where each p_i is the probability that the quantum system is in one of i states from a set $\{|\psi_i\}\rangle$ [16]. We can then represent the evolution of quantum states using operator-sum notation, which breaks a quantum operation (e.g., application of quantum noise that might—or might not—occur) into a summation that we can use to 'build up' descriptions of system-wide operations. Specifically, we can write,

$$\varepsilon(\rho) = \sum_k E_k \rho E_k^\dagger, \tag{9}$$

where $\varepsilon(\rho)$ is the system-wide quantum operation applying to system state ρ, and the E_k's represent matrices applying pieces of that quantum operation.

Quantum noise can thus be represented as a set of E_k's, meaning we can build a quantum operator ε to represent application of quantum noise. The use of density operators and operator-sum notation allows for representing quantum states in which there is uncertainty about whether or not noise will occur.

There are at least four types of quantum noise, all of which have different physical—and thus mathematical—representations. One general noise phenomenon is known as *decoherence*, which refers to loss of information when a qubit evolves towards a state that nature 'prefers' (e.g., a state where the qubit's temperature matches that of the environment) [16]. Two types of decoherence are dephasing noise and global depolarising noise. We focus on these here because they are the two types to which the VLS algorithm exhibits partial resilience in certain situations.

2. Dephasing Noise

Dephasing noise acting on a single qubit is mathematically represented in operator-sum notation with,

$$E_0 = \sqrt{p}\begin{pmatrix} 1 & 0 \\ 0 & 1 \end{pmatrix} = \sqrt{p}I \tag{10}$$

and

$$E_1 = \sqrt{1-p}\begin{pmatrix} 1 & 0 \\ 0 & -1 \end{pmatrix} = \sqrt{1-p}Z, \tag{11}$$

where p is the probability that the noise does not affect state ρ, I is the identity gate, and Z is the Pauli-Z gate [16].

Theorem 1. *If dephasing noise occurs at the end of the circuit, the VLS algorithm is resilient to that noise, because dephasing noise affects the off-diagonal elements of a quantum state, ρ, while the VLS algorithm stores computed information in the diagonals of ρ.*

Proof:

Corollary 1. *The VLS algorithm is resilient to single-qubit dephasing noise applied at the end of a quantum circuit.*

Proof: Considering the definition of dephasing noise as provided in eqs. (10) and (11), the density matrix of the dephasing noise operation, $\tilde{\rho}$ on a single qubit of a quantum state ρ is

$$\tilde{\rho} = \varepsilon_d(\rho) = pI\rho I^\dagger + (1-p)Z\rho \mathrm{Z}^\dagger, \tag{12}$$

. Here, p is the probability that dephasing noise does not disrupt ρ, while $(1-p)$ is the probability that it does. Simplifying gives,

$$\tilde{\rho} = p\rho + (1-p)Z\rho Z. \tag{13}$$

Thus, we see that ε_d will meaningfully change only the elements on the diagonal of ρ, given the second term in eq. (13). Because Z is diagonal with ± 1, it flips the signs of rows corresponding to the location of its -1 when it is left-multiplied with another matrix and flips the signs of columns corresponding to the same when it is right-multiplied with another matrix. Eq. (13) applies Z from both sides, meaning those sign flips will cancel each other out, leaving the diagonal elements of ρ unchanged.

Recall that the goal of the VLS algorithm is to estimate $|c_i|^2$ in $|x(\boldsymbol{\theta})\rangle = \sum_i c_i|\boldsymbol{z_i}\rangle$. Because $\boldsymbol{z_i}$ is the set of canonical basis vectors, we see that the terms involving $|c_i|$ are on the diagonal of the specified matrix. Thus, the most valuable information in our quantum state lies on the diagonal of the density operator representing that state:

$$\rho = |x(\boldsymbol{\theta})\rangle\langle x(\boldsymbol{\theta})| = \sum_i |c_i|^2|\boldsymbol{z_i}\rangle\langle\boldsymbol{z_i}| + \sum_{i\neq j} c_i c_j^*|\boldsymbol{z_i}\rangle\langle\boldsymbol{z_j}|, \tag{14}$$

given that the measurements are in the standard basis $\{|\boldsymbol{z_i}\rangle\}_{i=1}^{2^n}$ such that $\boldsymbol{z}_i \in \{0,1\}^{\otimes n}$.

Thus, as the diagonal elements of ρ have the relationships between them unchanged by dephasing noise acting on a single qubit, we see that the VLS algorithm has resilience to single-qubit dephasing noise occurring at the end of a circuit. □

We can now extend this argument to multiple qubits, by noting that the application of dephasing noise to each qubit at the end of a quantum circuit would have the form,

$$\varepsilon(\rho) = \varepsilon_n(\varepsilon_{n-1}(\ldots\varepsilon_1(\rho)\ldots)), \tag{15}$$

where ε_i is the noise applied to each of the i qubits. (Note that *all* of the noise must still be assumed to occur at the end of the circuit.) Then, $\varepsilon_1(\rho)$ has the form of the above corollary,

$$\varepsilon_1(\rho) = p_1\rho + (1-p_1)Z\rho Z. \tag{16}$$

Applying $\varepsilon_2(\rho)$ to the result of eq. (16) gives,

$$\begin{aligned} \varepsilon_2(\varepsilon_1(\rho)) &= p_2(p_1\rho + (1-p_1)Z\rho Z) \\ &\quad + (1-p_2)Z(p_1\rho + (1-p_1)Z\rho Z)Z \\ &= p_1p_2\rho + p_2(1-p_1)Z\rho Z \\ &\quad + p_1(1-p_2)Z\rho Z \\ &\quad + (1-p_1)(1-p_2)Z^{\otimes 2}\rho Z^{\otimes 2}. \end{aligned} \tag{17}$$

Similarly, we find that $\varepsilon_3(\rho)$ has the simplified form,

$$\begin{aligned} \varepsilon_3(\varepsilon_2(\rho)) &= p_1p_2p_3\rho + p_2p_3(1-p_1)Z\rho Z \\ &\quad + p_1p_3(1-p_2)Z\rho Z \\ &\quad + p_3(1-p_1)(1-p_2)Z^{\otimes 2}\rho Z^{\otimes 2} \\ &\quad + p_1p_2(1-p_3)Z\rho Z \\ &\quad + p_2(1-p_1)(1-p_3)Z^{\otimes 2}\rho Z^{\otimes 2} \\ &\quad + p_1(1-p_2)(1-p_3)Z^{\otimes 2}\rho Z^{\otimes 2} \\ &\quad + (1-p_1)(1-p_2)(1-p_3)Z^{\otimes 3}\rho Z^{\otimes 3}. \end{aligned} \tag{18}$$

We thus see that $\varepsilon_n(\rho)$ will have the form,

$$\varepsilon_n(\rho) = \tilde{\rho} = \sum_\mu q_\mu \boldsymbol{Z}_\mu \rho \boldsymbol{Z}_\mu, \tag{19}$$

where $\boldsymbol{Z}_\mu = \{I, Z\}^{\otimes n}$ because $Z^{\otimes n}$ for even n is I and for odd n is Z. Furthermore, the postulates of quantum mechanics require that $\sum_\mu q_\mu = 1$.

We can now consider eq. (19) in light of the solution stored by VLS. We illustrated in proving Corollary 1 that VLS stores information on the diagonals of the quantum state density operator it prepares. This means that the solution is stored in the measurement values $p(|z_i\rangle)$, so, to show that VLS exhibits resilience to dephasing noise applied to each qubit at the end of a circuit, we show that $p(|z_i\rangle)$ is the same whether or not dephasing noise is applied to one or more qubits.

First, we note that $p(z_i)$ in the case of dephasing noise applied to a single qubit is defined as,

$$p(z_i) = \langle z_i|\rho|z_i\rangle, \tag{20}$$

because the single-qubit noise leaves ρ unchanged. Similarly, in the case of dephasing noise applied to multiple qubits, $p(z_i)$ is defined as,

$$p(z_i) = \langle z_i|\varepsilon_n(\rho)|z_i\rangle = \langle z_i|\tilde{\rho}|z_i\rangle. \tag{21}$$

Plugging in the result of eq. (19) gives,

$$\begin{aligned} p(z_i) &= \langle z_i| \sum_{\mu} \boldsymbol{Z}_{\mu}\rho \boldsymbol{Z}_{\mu} q_{\mu}|z_i\rangle \\ &= \sum_{\mu} q_{\mu}\langle z_i|\boldsymbol{Z}_{\mu}\rho \boldsymbol{Z}_{\mu}|z_i\rangle. \end{aligned} \tag{22}$$

Here, as in the proof of Corollary 1, the dual $\boldsymbol{Z_{\mu}}$'s will cancel any effect on $\rho, \langle z_i|$, and $|z_i\rangle$. Furthermore, we stated that $\sum_{\mu} q_{\mu} = 1$, so eq. (22) simplifies to,

$$p(z_i) = \langle z_i|\rho|z_i\rangle, \tag{23}$$

which is precisely what it was for the case of single-qubit dephasing noise.

Therefore, as the diagonal elements of ρ have the relationships between them unchanged by dephasing noise applied at the end of a quantum circuit,the VLS algorithm is resilient to dephasing noise that occurs at the end of a circuit. □

3. *Global Depolarising Noise*

Global depolarising noise is caused by the tendency of the quantum state to shift towards a maximally-mixed state, which contains no relevant information beyond noise [16]. The maximally-mixed state is mathematically represented by $\frac{I}{2^n}$, where n is the number of qubits in the state. We can represent global depolarising noise mathematically as,

$$\tilde{\rho} = \varepsilon_p(\rho) = p\rho + \frac{(1-p)}{2^n}I, \tag{24}$$

where—as with dephasing noise—p is the probability that the state remains unaffected by global depolarising noise, and $(1-p)$ is the probability that ρ changes due to noise.

Theorem 2. *The VLS algorithm is partially resilient to global depolarising noise that occurs at the end of each circuit layer, because global depolarising noise does not change the sign information of the diagonal elements of the density operator, ρ, representing the VLS algorithm's prepared state.*

Proof: Any quantum circuit ansatz can be represented by a series of operations, each of which represents one of its unitary layers. Recall that each layer is a series of gates with identical gate type but varying parameters that the VLS algorithm seeks to tune. Let $U_l(\cdot)$ be one such ansatz layer of gates that run in parallel, and let $U(\boldsymbol{\theta})$ represent the entire ansatz, such that $U(\boldsymbol{\theta}) = \prod_{l=1}^{L} U_l(\boldsymbol{\theta})$, where L is the number of layers of gates, which scales linearly with the number of ansatz layers. So, the quantum state after each layer, $l = 1, ..., L$ can be represented as,

$$\rho_l = U_l\rho_{l-1}U_l^{\dagger}, \tag{25}$$

with ρ_{l-1} being the state immediately before application of layer l gates, $\rho_0 = |0\rangle^{\otimes n}\langle 0|^{\otimes n}$ being the initial state, and ρ_L being the final, resulting state.

Assume that global depolarising noise occurs after each layer. In other words, the quantum state $\tilde{\rho}_l$ after each layer and noise is

$$\tilde{\rho}_l = \varepsilon_p(\rho_l) = \varepsilon_p\left(U_l\rho_{l-1}U_l^{\dagger}\right). \tag{26}$$

Note that the initial state remains ρ_0 because we assume that no global depolarisation occurs until after the first ansatz layer (i.e., U_1).

Corollary 2. *Global depolarising noise scales exponentially with the number of layers while keeping its form*

$$\tilde{\rho}_l = p^l\rho_l + \frac{1-p^l}{2^n}I.$$

Proof: We can prove that global depolarising noise keeps the form given in Corollary 2 by induction. First, the base case $l = 0$ is true because—as stated above—the system has ρ_0 in both the situations of no global depolarising noise and global depolarising noise after each layer. And, $\tilde{\rho}_0 = p^0\rho_0 + \frac{(1-p^0)}{2^n}I = \rho_0$, which shows that $\tilde{\rho}_0$ can be correctly described using the form in Corollary 2.

So, we need next show the inductive step, namely that,

$$\tilde{\rho}_l = \varepsilon_p(U_l\tilde{\rho}_{l-1}U_l^{\dagger}) = p^l\rho_l + \frac{(1-p^l)}{2^n}I, \tag{27}$$

for all $l = 1, .., L$. Assume that the system after $1 \leq k - 1 \leq L$ layers and $k-1$ applications of global depolarising noise is given by

$$\tilde{\rho}_{k-1} = p^{k-1}\rho_{k-1} + \frac{1-p^{k-1}}{2^n}I. \tag{28}$$

Then, applying the next layer of gates and noise provides state

$$\tilde{\rho}_k = \varepsilon_p(U_k\tilde{\rho}_{k-1}U_k^{\dagger}). \tag{29}$$

Simplifying by plugging in eq. (28) for $\tilde{\rho}_{k-1}$ gives,

$$\begin{aligned} \tilde{\rho}_k &= \varepsilon_p(U_k(p^{k-1}\rho_{k-1} + \frac{1-p^{k-1}}{2^n}I)U_k^{\dagger}) \\ &= \varepsilon_p(p^{k-1}(U_k\rho_{k-1}U_k^{\dagger}) + \frac{1-p^{k-1}}{2^n}U_kIU_k^{\dagger}) \\ &= \varepsilon_p(p^{k-1}U_k\rho_{k-1}U_k^{\dagger} + \frac{1-p^{k-1}}{2^n}I). \end{aligned} \tag{30}$$

Applying the definition of ε_p from eq. (24) gives,

$$\begin{aligned}\tilde{\rho}_k &= p(p^{k-1}U_k\rho_{k-1}U_k^\dagger + \frac{1-p^{k-1}}{2^n}I) + \frac{(1-p)}{2^n}I \\ &= p^k U_k\rho_{k-1}U_k^\dagger + \frac{p(1-p^{k-1})}{2^n}I \\ &\quad + \frac{(1-p)}{2^n}I \\ &= p^k U_k\rho_{k-1}U_k^\dagger + \frac{(1-p^k)}{2^n}I.\end{aligned}$$

Finally, noting that eq. (26) is present in the above, we can simplify to,

$$\tilde{\rho}_k = p^k\rho_k + \frac{(1-p^k)}{2^n}I,$$

which is the form we sought to show for $1 \leq k \leq L$. Thus, Corollary 2 is proven. □

Having established the effect of global depolarising noise after each layer, we need only to establish that the values on the diagonal of the resulting density operator after layer i have the same sign as those in the non-noise-affected density operator after layer i (i.e., we need to compare elements on the diagonal of $\tilde{\rho}_l$ and ρ_l). Consider elements at indices aa and bb in $\tilde{\rho}_l$ such that $0 \leq l \leq K$, and $aa, bb \in \{00, 11, ..., (2^n-1)(2^n-1)\}$. Then, by the result of Corollary 2,

$$\begin{aligned}\tilde{\rho}_{l_{aa}} &= p^l\rho_{l_{aa}} + \frac{1-p^l}{2^n}, \\ \tilde{\rho}_{l_{bb}} &= p^l\rho_{l_{bb}} + \frac{1-p^l}{2^n}.\end{aligned} \tag{31}$$

We seek to show that the elements along the diagonal of $\tilde{\rho}_l$ have the same signs as the diagonal elements in ρ_l; this indicates that the sign information is preserved in the presence of global depolarising noise. We can illustrate this by considering the subtraction of $\tilde{\rho}_{l_{bb}}$ from $\tilde{\rho}_{l_{aa}}$, which gives,

$$\begin{aligned}\tilde{\rho}_{l_{bb}} - \tilde{\rho}_{l_{aa}} &= p^l\rho_{l_{aa}} + \frac{1-p^l}{2^n} \\ &- p^l\rho_{l_{bb}} - \frac{1-p^l}{2^n} \\ &= p^l(\rho_{l_{aa}} - \rho_{l_{bb}}),\end{aligned} \tag{32}$$

for all $l = 0, ..., L$. Because p^l is a probability value that is thus always positive, the above illustrates that the sign information on the diagonal elements of $\tilde{\rho}_l$ is the same as that on the diagonal elements of ρ_l. And finally, because the VLS algorithm stores the information we seek on the diagonal elements of the density operator (as proven for Theorem 1), then we see that the VLS algorithm is partially resilient to global depolarising noise because the sign of the information contributing to the solution is preserved in the presence of global depolarising noise after each layer of the circuit. □

B. 'Smart Encoding' to Reduce Complexity of Obtaining Solution

As discussed in the Introduction, quantum computers provide efficiency gains in *computing*, but not necessarily *obtaining*, problem solutions. We can motivate this with a straightforward example; consider the fracture network of approximately 10^{15} nodes that we considered at the beginning of the paper. A quantum computer will store the solution vector to a linear system using only about 50 qubits. However, those 50 qubits will represent a solution vector of approximately $2^{50} \approx 10^{15}$ elements. Furthermore, each of the elements in this desired solution vector is a probability, meaning a value between 0 and 1. So, our sought solution is 10^{15} values between 0 and 1.

When we take measurements from a quantum computer, we obtain individual solutions that allow us to form a probability distribution from which we can obtain the desired vector of probabilities. So, in a situation such as the 10^{15} nodes example, we can thus see that the number of measurements necessary to establish a probability distribution for an exponentially-increasing number of nodes between 0 and 1 is also going to grow exponentially: even if we needed only one measurement per node, we would require 10^{15} measurements.

Mathematically, we can show this as follows. Consider the probability amplitudes, $|c_i|^2$ for each of the i elements in the vector of solutions ($i = 0, ..., n-1$ for n nodes). These must sum to one, giving us that,

$$\sum_i |c_i|^2 = 1. \tag{33}$$

Therefore,

$$|c_i| \propto \frac{1}{\sqrt{n}}, \tag{34}$$

because there are n nodes. Therefore, each probability amplitude is exponentially small. Furthermore, taking N measurements, or 'shots,' definitionally provides a solution precision of $\frac{1}{\sqrt{N}}$. Therefore, to make $\frac{1}{\sqrt{N}}$ proportional to $\frac{1}{\sqrt{n}}$, N must be proportional to n, meaning that the number of shots we need grows exponentially.

In this paper, we sought to assess the performance of VLS, meaning we needed to obtain every element of the solution, meaning every probability amplitude, to see how well VLS computed each element. For this reason, we accepted the exponential cost. Therefore, we obtained information as shown for a simple example in Fig. 9. The grid represents the nodes in the fracture region, each of which is denoted with a letter (A through D). Each of the numbers in the nodes represents the index (in binary notation) of the element in the probability solution vector corresponding to the normalized pressure solution for that node. As shown in the vectors in Fig, 9, the number in each node is *also* the state (for each of the qubits, 1 and 2) whose probability is associated with the pressure for the desired node. So, measuring both of the qubits a

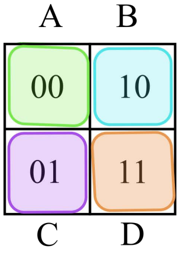

$$\begin{bmatrix} pressure_A \\ pressure_B \\ pressure_C \\ pressure_D \end{bmatrix} = \begin{bmatrix} prob_{1,2}(00) \\ prob_{1,2}(01) \\ prob_{1,2}(10) \\ prob_{1,2}(11) \end{bmatrix}$$

$$prob_{1,2}(00) = pressure_A$$
$$prob_{1,2}(10) = pressure_B$$

FIG. 9. **A 2×2 fracture network with measured pressures for each node and no 'smart encoding.'** Each of the nodes is labelled with a letter (A through D), and contains the index of the solution vector that will contain the pressure at that node. The vectors illustrate the probabilities that provide each of the node pressures, and the equations below provide two specific examples. First, the probability of measuring the first qubit to be 0 and the second qubit to be 0 provides the pressure solution for node A. Similarly, the probability of measuring the first qubit to be 1 and the second qubit to be 0 provides the pressure solution for node B.

number of times allows us to craft a probability distribution with one state probability per node.

While our approach was permissible for benchmarking the performance of the VLS algorithm, it is not sustainable for large fracture flow problems. Using exponential resources to obtain the solution would negate the advantage of quantum computing's speedup, and would become prohibitive for sufficiently large problems. Fortunately, the nature of fracture flow problems is such that one often desires the solution at only a subset of nodes. For situations in which this is not the case, however, we present 'smart encoding' as an alternative way to obtain aggregated information about more than one node without exponential complexity.

Consider Fig. 10, which presents a 'smart encoded' use of the situation in Fig. 9. Here, we can obtain information about a row or column of nodes by measuring just a single qubit, instead of measuring both.

While the benefits of smart encoding are limited in Fig. 10's trivial example, they become significantly more pronounced for larger problems. For example, consider larger regions in which information might be sought about certain pieces of that network. The desired solution could be the pressure in a *set* of nodes comprising a fracture, such as in Fig. 11, which presents a 16-node problem containing a pitchfork fracture. Here, if we arrange the indices in a thoughtful way, we can obtain information about the entire network when measuring only the first qubit. By determining the probability that the first qubit is zero, we learn the pressure throughout the non-pitchfork fracture region. Subtracting that value from one then provides the probability that the first qubit is one, which consequently provides the pressure throughout the pitchfork fracture. Future work should more fully consider both the benefits of and automated procedures for reformulating problems using smart encoding.

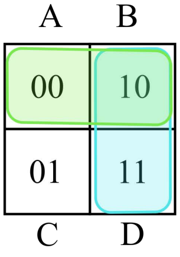

$$\begin{bmatrix} pressure_A \\ pressure_B \\ pressure_C \\ pressure_D \end{bmatrix} = \begin{bmatrix} prob_{1,2}(00) \\ prob_{1,2}(01) \\ prob_{1,2}(10) \\ prob_{1,2}(11) \end{bmatrix}$$

$$\begin{aligned} prob_2(0) &= prob_{1,2}(00) + prob_{1,2}(10) \\ &= pressure_A + pressure_B \\ prob_1(1) &= prob_{1,2}(10) + prob_{1,2}(11) \\ &= pressure_B + pressure_D \end{aligned}$$

FIG. 10. **A 2×2 fracture network using 'smart encoding.'** Again, each node is labelled with a letter (A through D), and contains the index of the solution vector that will contain the pressure at that node. While the vectors illustrating the probabilities that equate to each pressure are the same, the equations illustrate how we can measure just one of the qubits to obtain information about multiple nodes. First, the probability of measuring the first qubit to be 0 and the second qubit to be 0 provides the pressure solution for node A. Similarly, the probability of measuring the first qubit to be 1 and the second qubit to be 0 provides the pressure solution for node B.

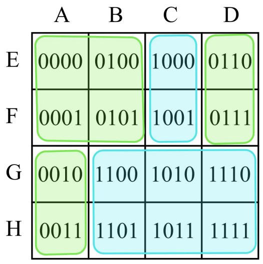

$$\begin{bmatrix} pressure_{AE} \\ pressure_{AF} \\ pressure_{AG} \\ pressure_{AH} \\ pressure_{BE} \\ pressure_{BF} \\ pressure_{DE} \\ pressure_{DF} \\ pressure_{CE} \\ pressure_{CF} \\ pressure_{CG} \\ pressure_{CH} \\ pressure_{BG} \\ pressure_{BH} \\ pressure_{DG} \\ pressure_{DH} \end{bmatrix} = \begin{bmatrix} prob_{1,2,3,4}(0000) \\ prob_{1,2,3,4}(0001) \\ prob_{1,2,3,4}(0010) \\ prob_{1,2,3,4}(0011) \\ prob_{1,2,3,4}(0100) \\ prob_{1,2,3,4}(0101) \\ prob_{1,2,3,4}(0110) \\ prob_{1,2,3,4}(0111) \\ prob_{1,2,3,4}(1000) \\ prob_{1,2,3,4}(1001) \\ prob_{1,2,3,4}(1010) \\ prob_{1,2,3,4}(1011) \\ prob_{1,2,3,4}(1100) \\ prob_{1,2,3,4}(1101) \\ prob_{1,2,3,4}(1110) \\ prob_{1,2,3,4}(1111) \end{bmatrix}$$

$$\begin{aligned} prob_1(0) &= prob_{1,2,3,4}(0000) + prob_{1,2,3,4}(0001) + prob_{1,2,3,4}(0010) + prob_{1,2,3,4}(0011) \\ &+ prob_{1,2,3,4}(0100) + prob_{1,2,3,4}(0101) + prob_{1,2,3,4}(0110) + prob_{1,2,3,4}(0111) \\ &= pressure_{AE} + pressure_{AF} + pressure_{AG} + pressure_{AH} + \\ &+ pressure_{BE} + pressure_{BF} + pressure_{DE} + pressure_{DF} \\ prob_1(1) &= prob_{1,2,3,4}(1000) + prob_{1,2,3,4}(1001) + prob_{1,2,3,4}(1010) + prob_{1,2,3,4}(1011) \\ &+ prob_{1,2,3,4}(1100) + prob_{1,2,3,4}(1101) + prob_{1,2,3,4}(1110) + prob_{1,2,3,4}(1111) \\ &= pressure_{CE} + pressure_{CF} + pressure_{CG} + pressure_{CH} + \\ &+ pressure_{BG} + pressure_{BH} + pressure_{DG} + pressure_{DH} \end{aligned}$$

FIG. 11. **A 4×4 pitchfork fracture network using 'smart encoding.'** This example illustrates how arranging the indices—and thus solution probabilities—in problem-specific ways can reduce the measurements required to obtain information about more than one node. Here, only the first qubit need be measured to differentiate the pressure throughout the pitchfork fracture from that in surrounding region.

C. Preliminary Results with Varying Permeability

After successfully scaling the 6x8 problem to larger region sizes, we returned to the smallest pitchfork problem to consider the affects of varying permeability. We once again embedded a pitchfork fracture in a region of lower permeability, but this time, the pitchfork contained

two permeabilities. Specifically, the right-most branch of the pitchfork had a permeability that was either 10 times, 100 times, 1000 times, or 10,000 times larger than the rest of the pitchfork. While the resulting system has the same size as the uniform-permeability pitchfork with five qubits, it is more complex because its elements vary by orders of magnitude more than in the uniform-permeability case.

Using the same general approach described in Sec. IV B, we obtained parameters for a parameterized quantum circuit that we then ran on the `ibmq_mumbai` machine. The training involved one specific procedural difference. Instead of training with forty instances of randomly-initialized parameters, we began with a set of trained parameters from earlier situations. For example, when training the circuit for a situation with a right-branch permeability of 10 times greater than the rest of the pitchfork, the initial parameters were those trained for the uniform-permeability pitchfork. And when training the circuit for a situation with a right-branch permeability of 100 times greater than the rest of the pitchfork, the initial parameters were either those trained for the uniform-permeability pitchfork or those trained for the right-branch-10-times-greater pitchfork.

We found that it was more difficult to obtain parameters that provided a high-fidelity result for these varying permeability problems than it was for the uniform-permeability variants. When running the best trained circuits on a classical simulator absent hardware noise, we achieved an average fidelity of 0.9555, to four digits of precision, illustrating that the classical optimization process had a more difficult time obtaining parameters than in the uniform-permeability-pitchfork case. Indeed, the fidelities suggest that each 10 times increase in permeability made optimal parameters harder for the classical optimization process to find. Specifically, although we were able to find parameters with a maximum 0.9808 fidelity for the situation with a rightmost-branch permeability of 10 times greater than the rest of the pitchfork, we found parameters with maximum fidelities of only 0.9763, 0.9757, and 0.9756 for problems with rightmost-branch permeabilities of 100 times, 1000 times, and 10,000 times greater than the rest of the pitchfork, respectively.

As expected, when we ran the circuits on the quantum hardware, noise reduced the fidelities achieved during training. Specifically, we achieved maximum fidelities of 0.9651, 0.9432, 0.9469, and 0.9467 for problems with rightmost-branch permeabilities of 10 times, 100 times, 1000 times, and 10,000 times greater than the rest of the pitchfork, respectively. Again, we used the `ibmq_mumbai` machine (qubits 0, 1, 4, 7, and 10) with 12 runs of 8192 shots each for a total of 98,304 shots. Fig. 12 illustrates results from the 10,000 times greater case.

While all of our experiments achieved fidelities greater than 0.9, the dip in accuracy demonstrates that there is room for improvement when it comes to solving more complex fracture problems with quantum algorithms. Fortunately, there are a number of forms such improvement could take, and here, we briefly discuss three.

First, we could further optimize the *classical* portion of the VLS algorithm. Classical optimization is complex in its own right, and we did not significantly tune the classical optimization process for the varying-permeability problems. The increased complexity of these problems suggests that such tuning might be worthwhile. Specifically, experimenting with which classical optimizer to apply [63] and how many layers of the ansatz to use might offer significant improvement, instead of applying the same choices that worked for uniform-permeability pitchfork cases. These considerations in particular might be especially fruitful because the final cost values for the trained circuits suggested that the optimizer was becoming stuck in a local minimum; alternative optimization methods specifically designed with that in mind might thus address this shortcoming.

Second, we could apply preconditioning methods to lower the condition-number of the matrix in the LSP prior to solving [47]. Ref. [47] suggests that reducing the condition number by applying methods specifically designed for solving LSPs with quantum algorithms can make the problem significantly less complex—and thus significantly less prone to error.

Third and finally, we might apply quantum error mitigation techniques. Quantum error mitigation is a relatively new field that is designed to address the shortcomings of near-term quantum hardware. Like variational algorithms, quantum error mitigation algorithms are designed for the NISQ hardware that we have today, and thus seek not to *correct* errors, but instead to *work around* them, in many cases by actually using the noise present, instead of solely trying to eliminate it [64]. For example, some quantum error mitigation approaches attempt to add selected gates to the circuit that will increase the amount of noise in such a way that the *net* amount of noise is reduced, due to interference between the 'automatically-present' noise and that added [64]. Another method known as zero-noise extrapolation supposes a given amount of noise present, increases that supposed amount of noise by a known factor greater than one, and then uses results with both noise levels to extrapolate what the results would be with a noise level of zero. [49, 64] In Ref. [51], zero-noise extrapolation contributed to significantly reducing error—in some cases by an order of magnitude—suggesting that the technique might provide at least some benefit were we to try it here.

Thus, our preliminary results for geologic situations involving multiple permeabilities within the pitchfork suggest that there is room for refinement when it comes to solving such problems with quantum algorithms and, specifically, with VLS. Fortunately, there are at least the three above readily-available avenues for such improvement.

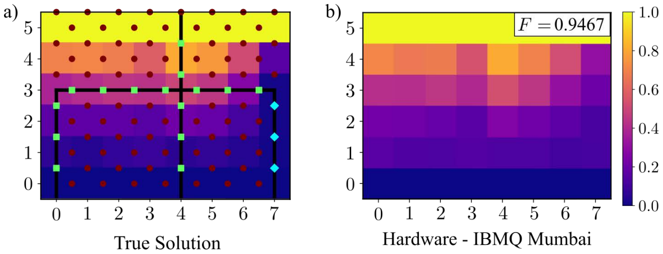

FIG. 12. **Solving a 6x8 Problem with Varying Pitchfork Permeability.** Subfigure (a) illustrates the known, classically computed solution with overlaid permeabilities. The blue diamonds are a permeability 10,000 times greater than that of the remainder of the pitchfork fracture, represented with green squares. The inner 4×8 nodes are the sought-after pressure values. Subfigure (b) is the solution from quantum hardware (specifically, qubits 0, 1, 4, 7, and 10 of the `ibmq_mumbai` machine). This solution has fidelity 0.9467, to four figures; the trained circuit achieved a maximum fidelity of 0.9756 when run on a noiseless classical simulator.
